# Books and Authors

**Published:** 1906-03

**Authors:** 


**International Clinics.** A Quarterly of Illustrated Clinical Lectures and especially prepared articles on Treatment, Medicine, Surgery, Neurology, Pediatrics, Obstetrics, Gynecology, Orthopedics. Pathology, Dermatology, Ophthalmology, Otology, Rhinology, Laryngology, Hygiene and other topics of interests to students and practitioners. By leading members of the medical profession throughout the world. Edited by **A. O. J. Kelly,** A.M., M.D., Philadelphia. Volume IV. Fifteenth series. 1905. Philadelphia and London: J. B. Lippincott Co. (Cloth, $2.00).

The fourth and last volume of the fifteenth series of these clinics is replete with valuable clinical facts and general material useful to the physician engaged in the active practise of medicine. Under the head of treatment are five articles; medicine, six; surgery, seven; obstetrics and gynecology, four; opthalmology, one; and pathology, two articles.

Among the articles, all of which are of merit, we desire to invite special attention to Dr. Deaver's, entitled "Results of Operations, such as Gastroenterostomy, Pyroplasty, etc., in the treatment of Diseases of the Stomach;" and to Dr. Wilcox's, entitled Medical Treatment of the Menopause." While we speak of these in particular, many of the other titles deserve praise, which must be omitted for want of space. The engravings are worthy of note and the entire book is an excellent number of this most excellent series.

---

**Proceedings of the Medico-Psychological Association** at the sixtieth annual meeting held at St. Louis, May 30-June 3, 1904.

This excellent volume of transactions of this scientific society is full of interesting material. It deserves a more durable cover in order to secure a better preservation of its contents. Dr. D.

R. Burrell, of Canandaigua, N. Y., the accomplished superintendent of Brigham Hall, one of the best institutions for the insane in the country, publishes an interesting case in sleep-talking. In the course of the article Dr. Burrell comments forcefully on the fact that much is written before the author emerges from the observing stage. Many interesting papers were read at the meeting and the association is to be congratulated upon its long and successful career.

---

**Gray's Anatomy, Descriptive and Surgical.** New American from the 15th English Edition. Revised, enlarged and rewritten by **J. Chalmers DaCosta,** M.D., professor of surgery in the Jefferson Medical College, in collaboration with a corps of specially selected assistants. In one imperial octavo volume of 1600 pages, with 1132 illustrations, 500 of which are new in this edition. Lea Brothers & Co., Publishers, Philadelphia and New York. 1905. (Price, with illustrations in black and many colors: cloth, $6.00 net; leather $7.00 net).

Notwithstanding the fact that Gray's Anatomy has been before the medical profession for fifty years, it still remains a leading work. It has kept pace with the advances in anatomical science during all these years, and each edition has represented the progress made in the study of human anatomy, by the ablest anatomists in both hemispheres. To John Chalmers DaCosta was committed the task of bringing out this new American edition, and everyone familiar with the profession on this side of the Atlantic will recognise the wisdom of the choice. The editor has revised every page, and has rewritten many sections such, for example, as those on the brain, spinal chord, nervous system, abdomen, and the lymphatics. The illustrations are superb, representing the highest type of the engraver's art. Five hundred of the eleven hundred and thirty-two engravings are new, while the whole number, both in color and in black and white, makes an understanding of the text easy, even next to actual dissection of the structures. It is a magnificent work and will ever remain a monument to its creator, Henry Gray, who died too young for the good of the world of medicine.

---

**A Manual of Diseases of Infants and Children.** By **John Ruhräh,** M.D., Clinical Professor of Diseases of Children, College of Physicians and Surgeons, Baltimore. 12mo volume of 404 pages, fully illlustrated. Philadelphia and London: W. B. Saunders & Co., 1905. (Flexible leather, $2.00 net).

This is most emphatically a manual for the medical pupil, and it is one of the best we have seen for that purpose. It may be regarded as an epitome of the subject. It embraces the best of all authors supplemented by the experience of the writer of the book. It does not waste words but states the pith of each topic. The advanced student will find this manual of vast usefulness in dealing with pediatrics for his finals. Moreover, it will serve him as a reference book after he begins practice. The text is a beautiful specimen of good printing and the illustrations are excellent.

**A Treatise on Diagnostic Methods of Examination.** By **Professor Dr. Hermann Sahli**, of Bern. Edited, with additions, by **Francis P. Kinnicutt,** M.D., Professor of Clinical Medicine, Columbia University, New York; and **Nathaniel Bowditch Potter,** M.D., Visiting Physician to the City Hospital and to the French Hospital; and Consulting Physician to the Manhattan State Hospital. New York, Philadelphia and London: W. B. Saunders & Co., 1905. Octavo of 1008 pages, illustrated. (Price: Cloth, $6.50 net; Half Morocco, $7.50 net).

The publication in English of this celebrated treatise has been awaited with interest by a large audience, ever since the announcement that this would be done. Now that the book has appeared great satisfaction is experienced in its treatment of the subject with which it deals. It is more than a work on diagnosis—it is also a clinical treatise in many respects. The author explains many clinical phenomena, physiologically and pathologically, even while considering diagnostic methods, hence it becomes by that much, the more valuable book.

Among the special features of Sahli's treatise are the directions given for the examination of the stomach contents, sputum, feces, urine, and blood. Never before have these important methods of investigating clinical facts been more clearly set forth or comprehensively stated. An article on blood-pressure by Janeway (T. C.) will attract attention because of growing importance attaching to this element in the diagnostic cycle.

Chemical and bacteriological methods of examination receive elaborate consideration, these laboratory methods being reduced to a simplicity which brings them within the purview of the clinician. The nervous system, too, receives comprehensive handling by the author. He makes plain many obscure facts that prove of diagnostic value. The fourth German edition, from which this book was translated, has received encomiums from the author's own countrymen, and it cannot be doubted that a similar reception will be given this translation by his American colleagues. The book deserves the most careful reading by every physician who desires to acquire accuracy in diagnosis.

---

**Modern Clinical Medicine.** Diseases of Metabolism, and of the Blood, Animal Parasites, Toxicology. Edited by **Richard C. Cabot,** M.D., instructor in Clinical Medicine in the Medical School of Harvard University. Authorised translation from "Die Deutsche Klinik," under the general editorial supervision of **Julius L. Salinger**, M.D. With one colored plate and 58 illustrations in the text. Octavo, pp. 650. New York and London: D. Appleton & Co. 1906. (Price: Cloth, $5.00).

In no branch of medicine has the literature been more enriched of late, than in the study of metabolism and of the diseases which its disturbances provoke. This volume constitutes the most important contribution that has yet appeared relating to this entire subject. The consumption of food in the healthy, and the requirements of food by the sick, are two questions that should receive more attention at the hands of physicians than is usually the case. Over nutrition and under nutrition are also two important points of study in relation to metabolism that should not be neglected. All these receive elaborate treatment in this book.

The diseases of metabolism,—diabetes mellitus, gout, obesity, myxedema, Addison's disease, acromegalia, chronic articular rheumatism, these and others,—are dealt with masterfully by specially trained clinical experts. In the section on diseases of the blood, we find detailed directions for blood examination which explain minutely all the processes of that important procedure. Then, diseases of the blood are considered, which include the anemias, chlorosis, leukemia, pseudo-leukemia, and the hemorrhagic diatheses. These are handled in a most satisfactory manner, every paragraph imparting information, and every sentence being full of meaning.

The animal parasites of man are taken up in the next section, and finally, the important poisons and their treatment are dealt with, the whole constituting the most important contribution which clinical medicine has received in recent years. Dr. Cabot's annotations as editor are in evidence enclosed in brackets, especially throughout the section relating to the blood. The translator has done an exceptionally clever piece of work in rendering the original text into smooth English. Every reader of the book will derive information that will be prove of lasting value.

---

**Operative Surgery for Students and Practitioners.** By **John J. McGrath,** M.D., Professor of Surgical Anatomy and Operative Surgery, at the New York Post Graduate Medical School; Surgeon to the Harlem,Post Graduate, and Columbus Hospitals;New York. Second revised edition, with 265 illustrations. Octavo, 628 pages. Sold only by subscription. Philadelphia: F. A. Davis and Company. 1905. (Price: Cloth, $4.50; half morocco, $5.50 net).

It is something less than three years since the first edition of this work was noticed in these columns. Advances have been made since then, and the exhaustion of that edition enables the author to add much new material embracing the changes of three years. While he drops part of the original title—Surgical Anatomy—he yet adheres to his former plan and blends anatomy and surgery in the descriptive text in a most instructive fashion. The surgery of the pancreas, spleen, prostate, and other viscera receive due attention, and the whole makes a book of great value to every surgeon.

---

**A Manual of Acute Poisoning.** . .With Methods for Use in First Aid to the injured. By **John W. Wainwright,** M.D. New York: E. R. Pelton, 1905. (Price, 75 cents).

This excellent manual gives classification, varieties, and individual substances usually met with in emergency poisoning, together with special symptoms, simple tests, chemical antidotes, physiologic anatagonists, and treatment in conditions of acute poisoning. In addition there is an excellent chapter supplying, in compact form, methods for use in first aid to the injured. Dr.

Wainwright has prepared a practical manual on an important subject, and has done it well. The size of the book is such that a physician may always have it with him without inconvenience.

---

**Transactions of the Southern Surgical and Gynecological Association.** Seventeenth session held at Birmingham, Ala., December 13, 14 and 15, 1904. **W. D. Haggard,** M.D., Secretary.

It requires a strong society to create each year a good volume of transactions. And yet, such a volume, no matter how good the material furnished, will not create itself. It is essential that an experienced eye and trained brain should supervise the work of putting the material through the press. No end of toilsome labor is required of such a man, and he sometimes gets naught but complaints for his tireless work, but he must do it, nevertheless; for the reputation of the society must be maintained no matter at what cost of time and labor. The volume before us is in many respects one of the best this association has issued, and testifies in the strongest manner to the indefatigable energy of its accomplished secretary. It contains among much interesting material, an account of the ceremonies of the unveiling of the monument erected to the memory of William Elias Brownlee Davis, the founder and long-time secretary of the association. A picture of the monument accompanies the record, and it is difficult to see how the association could have honored in a more befitting method the memory of our dearly beloved friend.

---

**Dissecting Manual,** based on Cunningham's Anatomy. By **W. H. Rockwell, Jr.,** M.D., Formerly Assistant Demonstrator of Anatomy in the College of Physicians and Surgeons, Columbia University, New York. Small octavo, pp. 306. New York: William Wood & Co. 1905. (Price: $2.00).

The division of the body into five parts, according to usual custom in the dissecting room, is followed in the descriptions given in this manual. First comes the head and neck; then the thorax; next the upper extremeties; followed by the abdomen and pelvis; and, finally, the lower extremeties These grand divisions are subdivided in a manner appropriate to their relations. The name of each muscle begins with a capital letter, with a view to catch the eye quickly, which, seems to us a good plan. Cunningham's Anatomy has been followed closely throughout the book, hence it will be specially appreciated wherever that work is used as a textbook. This useful manual will be appreciated by students of anatomy.

---

**Materia Medica and Pharmacy.** A Textbook for students of Medicine and Pharmacy, based upon the Fifth Edition of White & Wilcox's "Materia Medica and Therapeutics." By **Reynold Webb Wilcox,** M.A., M.D., LL.D., Professor of Medicine at the New York Post Graduate Medical School and Attending Physician to the Hospital, etc. etc.; Vice-Chairman of the Revision Committee of the U. S. P. Octavo. Philadelphia: P. Blakiston's Son & Co. 1905. (Cloth, $2.50 net).

This textbook is made to conform to the new U. S. pharmacopeia, which was issued in September 1905. While it is called the sixth edition it is in reality a new work, as it has been re-written in its entirety, to meet the changes which advances have caused. The list of therapeutic agents is divided into two main points,—inorganic and organic materia medica. The general classification is based on the grouping the substances according to the natural order to which each belongs. Professor Wilcox is thoroughly abreast of his subject, and his work is a reflex of long experience as a practical teacher of medicine. His position, too, on the Committee of Revision of the pharmacopeia lends additional weight to his statements regarding whatever pertains to materia medica and pharmacy.

